# Effects of Simplified Antihypertensive Treatment Algorithm on Hypertension Management and Hypertension-Related Death in Resource-Constricted Primary Care Setting between 1997 and 2017

**DOI:** 10.1155/2021/9920031

**Published:** 2021-07-13

**Authors:** Mulalibieke Heizhati, Nanfang Li, Qiaoyan Shi, Xiaoguang Yao, Delian Zhang, Keming Zhou, Menghui Wang, Junli Hu, Gulinuer Duiyimuhan, Wen Jiang, Jing Hong, Le Sun

**Affiliations:** National Health Committee Key Laboratory of Hypertension Clinical Research, Hypertension Institute of Xinjiang, Hypertension Center of People's Hospital of Xinjiang Uygur Autonomous Region China, No. 91, Tianchi Road, Tianshan District, Urumqi 830001, Xinjiang, China

## Abstract

Hypertension management is poor in primary care settings of developing countries, where 75% of hypertensives are living. Exploring better ways to improve hypertension management and to decrease stroke and CVD death is needed such as introducing treatment algorithm. Therefore, we selected intervention counties from Xinjiang, an underdeveloped region in China, and introduced antihypertensive treatment algorithm, comprising locally available and affordable agents, to primary health providers since 1998. Program effects were evaluated using the data collected in various ways including cross-sectional screenings to population ≥30 years between 1998 and 2015 by comparing treatment and control rates of hypertension, changes in blood pressure (BP) levels and distribution, and proportion of case/total and NCD death for CVD and stroke. Compared to 1998–2000, treatment rate was improved by 2.78 fold (11.2% vs. 32.1%, *P* < 0.001), and the overall and treated control rate were improved by 53.5 fold (0.2% vs. 10.7%, *P* < 0.001) and by 16.8 fold (2.0% vs. 33.5%, *P* < 0.001), respectively, in 2015. Mean SBP and DBP showed a net reduction by 33.7 mmHg (181.3 vs. 147.6 mmHg) and 21.3 mmHg (106.3 vs. 85.0 mmHg), respectively, in 2015, compared to 1998–2000 (*P* < 0.001), and stage III hypertension was reduced by 75.2% (33.5 vs. 8.3%, *P* < 0.001). Compared to 1997–1999, stroke/NCD death was reduced by 34.1% in 2015–2017 (31.7 vs. 20.9%, *P* = 0.006) in the intervention counties whereas by 7.5% in control county. Introduction of treatment algorithm helps improve hypertension management and reduce stroke death in resource-constricted primary settings.

## 1. Introduction

Cardiovascular diseases (CVD), responsible for 30% of all deaths at the time, became the leading cause of death worldwide as of the mid-1990s [1–3], and the burden was more huge in developing countries. Based on the first-generation global burden-of-disease study in 1990, 57.5% (3.6 million in total 6.26 million) deaths from ischemic heart disease and 68.5% (almost 3 million in total 4·38 million) deaths from stroke, the first and second most common causes of death among 30 leading causes of global mortality, took place in the developing regions [2]. In addition, mortality from ischemic heart disease in developing countries was expected to increase by 120% for women and 137% for men between 1990 and 2020 [4].

Increasing prevalence of hypertension and its poor control were the important factors in the epidemic of CVD in low- and middle-income countries [5–7]. For instance, in 2000 about 26.4% of adult population had hypertension, estimated to be 972 million, of whom 65.7% lived in economically developing countries with poor control rates [8]; in rural Ecuador, typical of developing countries, control of hypertension in early 2000 was as low as 0.3% [9].

An epidemic of heart disease and stroke may be inevitable for the developing world in lieu of early action, and the only hope to blunt its impact is to understand its origin and to control risk factors including hypertension, where its contribution is high. However, high illiteracy rates, poor access to health facilities, poverty and high costs of drugs characterized the status of hypertension in developing countries and contributed greatly to poor BP control [6]. Scarce resources and projected importance of hypertension and its future effects are important stimuli for innovative solutions that could help to override enormous difficulties of these deficient health systems. In this process, primary care staff, who are generally not hypertension focused, are partners in this enterprise and are part of the solution [10, 11]. A cost-effective approach is recommended to be put in practice to achieve these goals [12]. To manage hypertension, developing countries may need a simple algorithm for treatment, a reliable drug supply, affordable drugs, and mortality surveillance, whereas there was not sufficient evidence at the time [13]. Therefore, we explored a program directed at primary care providers and involved in simplified treatment algorithm at three counties of Xinjiang China, representative of developing regions, between 1998 and 2015, where historically hypertension was affecting one in two (51.9%) of population aged ≥30 years in 1997–1998 [14], disease burden resembles that of developing regions, and mortality from ischemic cerebrovascular disease, ischemic heart disease, and hypertensive heart disease is higher than national average [15]. The program was followed by evaluation using the data generated in various ways including cross-sectional surveys and death surveillance. Objective was to improve hypertension management and to decrease mortality due to stroke and CVD.

## 2. Methods

### 2.1. Site Selection

Intervention and control counties, located in Northern Xinjiang and home for mainly Han, Kazakh, and Mongolian ethnic groups, were selected using multistage stratified sampling method in 1998. In the first stage, Northern Xinjiang was stratified into five regions based on its administrative division as Aletai, Tacheng, Yili, Changji, and Bole regions. Three regions, Aletai, Tacheng, and Changji, were selected using simple random sampling (SRS) method. In the second stage, one to two counties were selected using SRS: Fuhai from Aletai, Hefeng and Tuoli from Tacheng, and Fukang from Changji. In the third stage, the four counties were divided into intervention (Fuhai, Hefeng, and Fukang) and control (Tuoli) area. Control area was not involved in program introduction, whereas collection of death data was conducted to compare changes in case/total death and case/noncommunicable chronic diseases (NCD) death for stroke and CVD over time.

## 3. Development of Evidence-Based Treatment Algorithm

There was a gap between existing knowledge and clinical practice at primary care setting of developing countries. In order to bridge the gap, we developed an antihypertension treatment algorithm.

### 3.1. Rationale for the Treatment Algorithm and Agent Selection

As in the algorithm in the supplementary figure, we developed a treatment algorithm, with the integrative BP control mechanisms in mind including reduction of sodium and water retention by diuretics, renin angiotensin aldosterone system (RAAS) inhibition by angiotensin converting enzyme inhibitors (ACEI), vasodilation by dihydropyridine calcium channel blockers (CCB) and controlling cardiac rate and output by nondihydropyridine CCB and beta-blockers. In fact, drug regimens with complementary activity, where a second agent blocks compensatory responses to the initial agent or affects a different pressor mechanism, can result in additive BP lowering. For example, diuretics may stimulate RAAS and an additive BP lowering effect may be obtained by adding an ACEI. In addition, in order to help accelerate uptake of the algorithms and clinical decision making, one specific agent, hydrochlorothiazide, captopril, nitrendipine, verapamil, and metoprolol, was selected, based on the fact that they were made available by the local government who rectified and guaranteed sustainable procurement mechanism and affordability. Efficacy and safety of verapamil in this algorithm were tested in smaller local sample [16]. Moreover, verapamil and metoprolol were prescribed for hypertensives with pulse rate ≥80 beat per minute (bpm), which can be simply done by calculating; thus it might be more suitable to the condition of limited doctors with medical license and equipment.

### 3.2. Application of the Treatment Algorithm to Patients

The first step is defining hypertension. The second step, diuretics were prescribed for 14 days. The third step, if target BP was not reached within 14 days, ACEI or dihydropyridine CCB was added for another 14 days. The fourth step, after ACEI prescription, if BP was still ≥140/90 mmHg, verapamil (nondihydropyridine CCB) for those with pulse rate ≥80 bpm and dihydropyridine CCBs for those with pulse rate <80 bpm were added for the following 14 days. After the prescription of dihydropyridine CCBs at the third step, if BP was still higher than the target level, beta blocker for those with pulse rate ≥80 bpm and ACEIs for those with pulse rate <80 bpm were added for the following 14 days. If BP reached the target level at any phase, patients would be followed up at every 3 months.

If BP was still higher than the target level after prescribing three BP-lowering agents, patients should be referred to next level hospital. If there is/are a previous heart attack, atrial fibrillation, symptoms/signs of heart failure, known heart failure, chronic kidney disease, symptoms/signs of stroke, known stroke, diabetes, or breast-feeding women, pregnant women, women of childbearing age, or BP > 200/120 mmHg, BP > 180/110 mmHg with severe headache, chest pain, breath shortness, blurred vision, vomiting, referral to next level hospital was performed. Health providers were encouraged to follow the algorithm unless clinical discretion was required for above conditions. For managing known hypertension, health care providers were advised to review medication and BP, returning to the antihypertensive treatment algorithm the program recommended. If BP was controlled to the target level and the medications were locally accessible and affordable, there was no reason to change the regimen. Recommended target BP was <140/90 mmHg for most patients to keep the intervention simple for both patients and health care providers.

## 4. Introduction of the Algorithm

Education program was initiated to primary health providers and conducted at each county annually since 1998. Primary health providers including those with and without medical license were educated on the algorithm. While conducting educational programs, local governments were involved by distributing official statement and by coordinating efforts from health bureaus, which were responsible for organizing annual educational programs, gathering health providers, and providing specific sites for program. Specialists from Hypertension Institute of Xinjiang were responsible for preparing training materials (slices, hand books, pocket cards, and wall posters of treatment algorithm) and for performing trainings which were at least 8 hour refresh workshop each year at each county followed by on-site clinical case-based training. Considering multiethnic background, materials were prepared and trainings were conducted in 4 languages. All health providers who participated in the training were assessed for uptake of the training contents by before-after-training examinations yearly [17–19].

## 5. Evaluation

### 5.1. Parameters

#### 5.1.1. Comparison of Changes in Treatment and Control Rates, BP Levels, and Hypertension Stage

To evaluate overall effects of the program, treatment, and control rates of hypertension, control rate in the treated, BP levels and percentage of hypertension stages were compared using the data from cross-sectional surveys conducted in 1998–2000, 2007–2008, and in 2015.

#### 5.1.2. Comparison of Proportion of Case/Total Death and of Case/NCD Death for Hypertension-Related Disease Over Time

To assess influence of the program on mortality, proportion of case/total death and of case/NCD death for CVD and stroke were compared in 1997–1998, 2007–2009, and in 2015–2017 using before-after comparison.

#### 5.1.3. Comparison of Death Data with the Control Area

We also compared proportion of case/total and NCD death between Tuoli and other three counties during the same period in order to assess secular trends of mortality in control and intervention area.

## 6. Methods

### 6.1. Cross-Sectional Surveys

Cross-sectional surveys were conducted with approval from Ethics Committee of People's Hospital of Xinjiang Uygur Autonomous Region. Independent cross-sectional surveys were performed in Hefeng (1998, 2008, and 2015), Fuhai (2000 and 2015), and Fukang counties (2007 and 2015).

### 6.2. Concrete Methods

Methods were similar for all surveys as described elsewhere [20]. In brief, at least 2 days prior to the survey, all inhabitants aged ≥30 years were informed by trusted local government officials and local health providers in order to increase attendance rate of survey contents. After signing the informed consent on-site, subjects were enrolled. Subjects were offered advice on healthy life styles and on medical seek if needed. Also, one time antihypertensive medications were offered to hypertensives who were willing to accept the treatment or referred to related health care centers. All methods were performed in accordance with the Declaration of Helsinki.

### 6.3. Data Collection

Data were collected by physicians and trained nurses, based on study protocol using a standard questionnaire in face-to-face interviews. Information was on demographic data (age, sex, and ethnicity), education, lifestyle-related factors (cigarette and alcohol consumption), and hypertension-associated parameters (if the condition was previously diagnosed by a physician and whether antihypertensive agents were taken for the last two weeks). Physical examination was conducted, including BP measurement. Three BP measurements were taken using a mercury sphygmomanometer via standardized procedures after a rest of at least 5 min from the unclothed right arm of the person in a sitting position at intervals of at least 1 min. For the purposes of analysis, the mean of the three measurement was considered. Body weight, height, and waist circumference were measured using standard methods.

Data on death records were collected from the household registration system, hospital records, and health insurance system in 1997–1998, in 2007–2009, and in 2015–2017 from all sites, and the death diagnosis was based on the death certificate.

### 6.4. Definitions

Hypertension was defined as systolic BP (SBP) ≥ 140 mmHg or diastolic BP (DBP) ≥ 90 mmHg, previous physician-diagnosed hypertension, and/or the use of antihypertensive medication within the prior two weeks. Treatment of hypertension was defined as use of antihypertensive medication in the past two weeks. Control of hypertension was defined as BP < 140/90 mmHg after taking antihypertensive medications. Control in treatment was defined as the percentage of those on treatment with BP < 140/90 mmHg among total hypertensives under treatment. Hypertension grading was stage I: SBP 140–159 mmHg and or DBP 90–99 mmHg; stage II: SBP 160–179 mmHg and or DBP 100–109 mmHg; stage III: SBP ≥180 mmHg and or DBP≥110 mmHg using the Chinese hypertension guideline [21]. Awareness of hypertension was described as self-report of any previous physician-diagnosed hypertension. Current cigarette consumption was defined as those who smoked at least one cigarette per day and had lasted for at least 6 months. Current alcohol intake was defined as those who drank at least twice per month and had lasted for at least 6 months.

CVD is the name for a group of disorders of heart and blood vessels, which includes hypertension, coronary heart disease, cerebrovascular disease (stroke), peripheral vascular disease, heart failure, rheumatic heart disease, congenital heart disease, and cardiomyopathies [22]. Stroke included ischemic and hemorrhagic stroke in the current study [23]. Common NCD included CVD, cancer, gastrointestinal, respiratory, renal, hepatic, and neurological diseases and injury [24].

### 6.5. Statistical Analysis

Continuous variables including BP, age, and body mass index (BMI) were expressed as mean ± standard deviation (SD) if normally distributed, comparison among groups was performed using ANOVA method, and 95% confidence intervals (CI) were calculated. The categorical variables, including gender, age groups, ethnic composition, education level, and smoking and drinking status were summarized as *n* (%) and compared using X^2^ test among groups. Data on prevalence of hypertension were standardized for age, gender, and ethnicity using the current study subjects in 1998–2015. Proportion of case/all-cause mortality for CVD and stroke was calculated, expressed as % and compared using *X*^2^ test among groups. Data were inputted to Excel Software by professionals and analyzed using the Statistical Program for Social Sciences, version 19.0 (SPSS Inc., Chicago, IL, USA).

## 7. Results

### 7.1. Population Characteristics

Table 1 shows characteristics of study population between 1998 and 2015. Totally 6143 adults aged ≥30 years participated in surveys (3543 women, 57.7%). Complete data were obtained from 1,551 (96.4%) in 1998–2000, from 2330 (96.6%) in 2007–2008, and from 2262 (93.3%) subjects in 2015. Percentage of subjects with senior high and higher education was lower in 2015 than in 1998–2000 and in 2007–2008. Proportion of cigarette consumption was 25.5% in 1998–2000 and 18.1% in 2015.

### 7.2. Changes in Age-Gender-Ethnicity-Standardized Treatment and Control of Hypertension between 1998 and 2015

**Figure 1:**
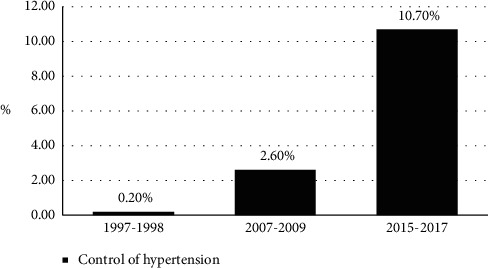
Changes in control of hypertension over time.

As given in Table 2 and Figure 1, treatment, and overall control and treatment control rates of hypertension improved over time. Compared to 1998–2000, treatment rate was improved by 2.78 fold (11.2% vs. 32.1%, *X*^2^ = 101.86, *P* < 0.001) in 2015. Compared to 1998–2000, control rate improved by 53.5 fold in all hypertensives (0.2% vs. 10.7%, *X*^2^ = 120.85, *P* < 0.001) and improved by 16.8 fold in treated hypertensives (2.0% vs. 33.5%, *X*^2^ = 59.46, *P* < 0.001) in 2015. In addition, compared to 1998–2000, awareness rate of hypertension in 2015 was improved by 59.6% (33.4% vs. 53.3%, *X*^2^ = 85.29, *P* < 0.001) in 2015.

#### 7.2.1. Changes in BP and Hypertension Stages between 1998 and 2015

**Figure 2:**
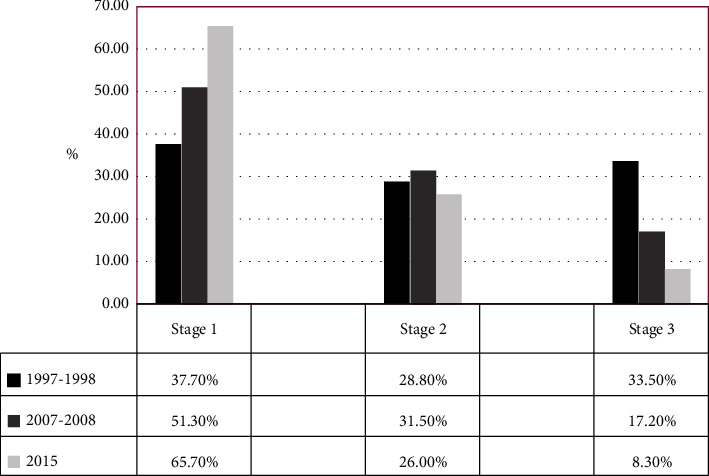
Changes in percentage of hypertension stages in 1997‐2015.

As in Table 3, mean of SBP and DBP decreased in total population over time. Reduction in SBP and DBP was the most obvious in hypertensives who received treatment. Mean SBP and DBP showed a net reduction by 33.7 mmHg (181.3 vs. 147.6 mmHg) and 21.3 mmHg (106.3 vs. 85.0 mmHg), respectively, in 2015, compared to 1998–2000 (*F* = 58.59, *P* < 0.001). Furthermore, proportion of stage I hypertension was increased by 42.6% (37.7 vs. 65.7%) and that of stage III hypertension was reduced by 75.2% (33.5 vs. 8.3%) in 2015, compared to 1998–2000 (*X*^2^ = 177.26, *P* < 0.001) as in Figure 2.

#### 7.2.2. Changes in the Proportion of Case/NCD Death for Stroke and CVD

**Figure 3:**
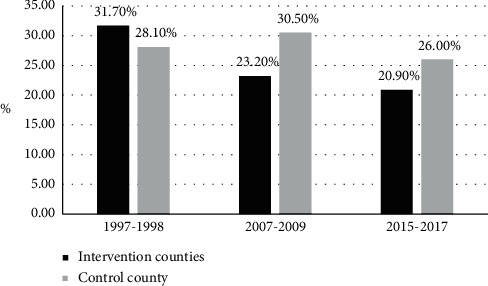
Changes in the proporton of case/NCD death for stroke between 1997 and 2017.

**Figure 4:**
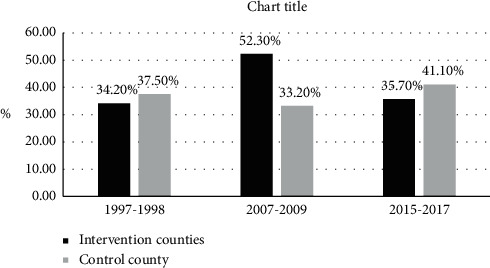
Changes in the proporton of case/NCD death for CVD between 1997 and 2017.

Proportion of case/NCD death showed a decreasing trend for stroke over time. Compared to 1997–1999, it was reduced by 34.1% in 2015–2017 (31.7 vs. 20.9%, *X*^2^ = 10.29, *P* = 0.006) in intervention counties; in control county, the proportion also showed decreasing trend and was reduced by 7.5% (28.1% vs. 26.0%), whereas it was not significant as in Figure 3. In intervention counties, proportion of CVD/NCD death showed an increase between 1997 and 2009, but then there was a significant reduction after that from 52.3% to 35.7% (*X*^2^ = 43.57, *P* < 0.001). Nonetheless, it showed a slight steady insignificant increasing trend in control county (37.5% vs. 41.1%) between 1997 and 2017 as in Figure 4.

## 8. Discussion

Hypertension is a key driver of CVD and remains widely undetected, undertreated, and poorly controlled in developing countries [25]. We introduced a treatment algorithm in a resource-constricted less developed region since late 1990s to build capacity in primary health providers, improve hypertension management, reduce hypertension-related death, and provide examples for settings with similar conditions.

During a period of 17 years, treatment in hypertension and control rate in total and in treated hypertensives were improved by 2.78,50 and 26.8 fold, respectively; mean SBP and DBP showed a net reduction by 33.7 mmHg and 21.3 mmHg in treated hypertensives. More importantly, proportion of stroke/NCD death was reduced by 34.1% in intervention counties, greater than that of 7.5% in control area. These improvements possibly indicate that introduction of treatment algorithms may have been helpful in capacity building, improving hypertension management, and reducing stroke mortality in a low-income primary care setting with insufficient medical resource.

In baseline survey, hypertension treatment and control in total and in treated patients were 11.1%, 0.2%, and 1.2%, respectively, lower than national average (control:1.2% in 1991, treatment: 20%, control: 5%, and control in treatment: 24% in 2002 [26, 27]), even though there is disparity in ages (≥18 years in national data) and screening methods of studied population. Theoretically, study population who are older and screened like ours should have had higher treatment and control rates due to increased possibility of seeking medical help with aging and increased awareness. Control rate is 0.2% in our data in 1998–2000, approximate to 0.3% of rural Ecuador at the time [9]. After 17 years of the program, treatment and control rates were 32.5% and 10.9% in our subjects, higher than 22.9% and 5.7% of adults aged 35–75 years between 2014 and 2017 in China PEACE project with similar sampling method with ours [28]. In addition, control rate in treated hypertensives is higher than that of random-sampled subjects aged ≥35 years from Xinjiang in Cardiovascular Risk Survey (32.1% vs. 24.3%) [29]. Supportive of current results, Tocci et al. evaluated trends in BP and awareness, treatment, and control rates of hypertension among adults visiting open checkpoints in Italy from 2004 to 2014 at annual World Hypertension Day and observed that awareness increased over time and control in treatment increased from 50.0% in 2004–2010 to 57.6% in 2013–2014 [30].

This paper provides a suggestion that real-life education program in primary care setting over the years shows improved treatment and overall and treated control rates of hypertension in Xinjiang, China. The primary aim was capacity building among primary care providers including those with and without medical license by introducing treatment algorithm. Presence of the algorithm with specific evidence-based, locally available, and affordable agents may have helped address barriers to hypertension control related to health providers such as medication selection and therapeutic inertia [31]. In addition, rational combinations may have been helpful to improve synergistic effects and to minimize side effects [32]. Furthermore, almost all hypertensives may not have to use out-of-pocket pays since selected medications were covered by medical insurance system, with new rural medical cooperative care system since 2003, which may have also helped address patient-related barriers such as economical factors [33]. The algorithm contains some differences with current guidelines. First, we selected diuretics as the first step agent since diuretic, thiazide type is superior to CCB and ACEI in preventing CVD in general population [34, 35]; in terms of lifestyle preferences and traits, locals are prone to high salt intake [31]. Second, most current guidelines recommend dual therapy with fixed dose combinations, whereas we selected free low-dose combinations. Third, we considered the easy-to-get and simple-to-use natures of the algorithms; those who can measure BP and calculate pulse rate can use the algorithm.

During the time, proportion of stroke/NCD death reduced by 34.1%, 4.5 fold greater than the 7.5% reduction in control area. Introduction of the algorithm may have been followed by decreased stroke mortality possibly due to better out-of-hospital BP control and reduction in stroke incidence [32] within this specific time frame, since >70% of stroke mortality in China can be explained by hypertension [36]. In fact, it is shown that better out-of-hospital BP control has a positive effect on stroke mortality and vice versa [37]. In addition, current results are consistent with the fact that age-standardized death rate fell by 25.7% for total NCDs including stroke between 1990 and 2013 in China [15], although the proportion of stroke/NCD mortality is not as sensitive as stroke mortality per se.

In this data, we observed that SBP and DBP levels of treated hypertensives were higher than those of the untreated, possibly attributable to the following reasons. First, older subjects characterized by arteriosclerosis and elevated SBP, with longer hypertension duration or with more comorbidities, are more prone to take antihypertensive agents, specially decades ago, whereas their BP levels are difficult to reach target. In our sample, antihypertensive takers were older (57.1 vs. 48.0 years, *P* < 0.001) and had larger BMI (28.2 vs. 25.8 kg/m^2^, *P* < 0.001), compared with the untreated (data not given). In addition, >70% hypertensives need ≥2 agents to reach target BP [38], whereas almost 90% hypertensives from Xinjiang were taking single agent in 2012–2015, based on our another survey [39], which may be even more worse decades ago.

This study has both strengths and limitations. It was strengthened by careful baseline evaluation of hypertension burden locally and random site selection, long duration, and population-based cross-sectional surveys for evaluation. In addition, we included a control county to evaluate program effects on hypertension-related mortality. Nonetheless, it should also be admitted and kept in mind that there are several limitations. First, there are other factors contributing to improvements in hypertension awareness, treatment, and control. For instance, local socioeconomic conditions have shown significant improvement (GDP: 111.66billion RMB in 1998, and 932.48 billion RMB in 2015 [40]); population acquiring higher education increased from 19.0% in 2000 to 24.3% in 2012 [41, 42]. Second, NCD prevention and control have been on national policy agenda, followed by tremendous increases in coverage and utilization of health care resources [43]. Third, due to cross-sectional survey over time, we cannot provide causation between the program and improved management of hypertension, but just relevance. Fourth, due to on-site measurement of BP, we were unable to exclude the potential bias of white coat effect, whereas this data were compared with each other over time, which may have offset the bias. Fifth, we collected death retrospectively, and thus we failed to acquire exact information on some of the important parameters, which made it difficult to estimate the event rates. Sixth, although parameters of hypertension management showed some improvements over time, percentage of cigarette consumers, alcohol takers, and the overweight and obese also increased. This may imply the significance of highlighting healthy life style, which may further optimize hypertension management and further reduce CVD burden.

In conclusion, introduction of treatment algorithm may help improve hypertension management and reduce stroke death in low-income medical resource-constricted primary care settings, referred by settings with approximate conditions.

## Figures and Tables

**Table 1:** Characteristics of study population by years of the surveys.

	1998–2000 (*n* = 1551)	2007-2008 (*n* = 2331)	2015 (*n* = 2262)	*X* ^2^/*F*/*P* value
Gender (women, *n*, %)	933 (60.1)	1390 (59.7)	1220 (53.9)	20.62/<0.001
Age (years)	47.5 ± 11.5	48.1 ± 10.8	50.6 ± 13.3^bc^	39.71/<0.001
Age group (*n*, %) 30–39	419 (27.0)	566 (24.3)	534 (23.6)	98.83/<0.001
40–49	518 (33.4)	785 (33.7)	646 (28.6)	
50–59	348 (22.4)	604 (25.9)	481 (21.3)	
≥60	266 (17.2)	376 (16.1)	601 (26.6)	
Ethnicity (*n*, %) Kazakh	1041 (67.1)	1311 (56.2)	507 (22.4)	1142.24/<0.001
Han	103 (6.6)	482 (20.7)	1102 (48.7)	
Mongolian	407 (26.3)	538 (23.1)	653 (28.9)	
BMI (kg/m^2^)	25.1 ± 4.2^ab^	26.6 ± 4.3^c^	26.1 ± 4.3	53.56/<0.001
BMI ≥24 kg/m^2^ (*n*, %)	911 (22.1)	1658 (40.2)	1552 (37.7)	68.59/<0.001
Education (*n*,%) ≤primary	861 (55.5)	1007 (43.2)	1361 (60.2)	266.96/<0.001
Junior high	381 (24.6)	596 (25.6)	626 (27.7)	
≥Senior high	309 (19.9)	728 (31.2)	275 (12.2)	
Cigarette consumption (*n*, %)	396 (25.5)	318 (13.6)	409 (18.1)	644.08/<0.001
Alcohol intake (*n*, %)	353 (22.8)	358 (15.4)	477 (21.1)	672.60/<0.001
Presence of hypertension	877 (56.5)	1133 (48.6)	662 (29.3)	310.33/<0.001

BMI: body mass index. 1998–2000: Hefeng and Fuhai; 2007-2008: Fukang and Hefeng; 2015: Hefeng, Fuhai, and Fukang. ^a^1998–2000 vs. 2007-2008, ^b^1998–2000 vs. 2015, and ^c^2007-2008 vs. 2015.

**Table 2:** Changes in awareness, treatment, and control of hypertension between 1998 and 2015 (*n*, %).

	1998–2000		2007-2008		2015		*X* ^2^/*P* value
	*n*	% (95% CI)	*n*	% (95% CI)	*n*	% (95% CI)	
Awareness	293	33.4 (30.3,36.5)	576	50.8 (47.9,53.8)	353	53.3 (49.5,57.1)	85.29/<0.001
Treatment	98	11.2 (9.1,13.3)	263	23.2 (20.8,25.7)	215	32.1 (28.9,36.1)	101.86/<0.001
Control 1	2	0.2 (0.1,0.5)	30	2.6 (1.7,3.6)	72	10.7 (8.5,13.3)	120.85/<0.001
Control in the treated	2	2.0 (0.8,4.9)	30	11.4 (7.5,15.3)	72	33.5 (27.1,39.8)	59.46/<0.001
Control 2	1	0.1	12	1.1	34	5.1	59.84/<0.001

*n*: the number of hypertensives with awareness, under treatment, with blood pressure controlled. Control 1 was defined as BP < 140/90 mmHg; control 2 was defined as BP < 130/80 mmHg.

**Table 3:** Trends in blood pressure in study population between 1998 and 2015 (mmHg).

	1998–2000	2007-2008	2015	F/*P* value
	Mean (95% CI)	Mean (95% CI)	Mean (95% CI)	
*Total population*				
SBP	144.4 (142.9, 145.9^)ab^	133.7 (132.7, 134.7)^c^	127.7 (126.8, 128.5)^abc^	209.45/<0.001
DBP	89.6 (88.8, 90.4)^ab^	85.8 (85.2, 86.4)^b^	76.4 (76.0, 76.9)^ab^	466.35/<0.001

*Hypertensives treated*				
SBP	181.3 (175.4, 187.3)^ab^	161.1 (157.8, 164.5)^c^	147.6 (144.8, 150.4)^abc^	58.59/<0.001
DBP	106.3 (102.7, 110.0)^ab^	98.3 (96.4, 100.3)^c^	85.0 (83.2, 86.8)^abc^	77.90/<0.001

*Hypertensives untreated*				
SBP	161.4 (159.6, 163.2)^ab^	148.7 (147.4, 150.0)^c^	153.0 (151.5, 154.4)^abc^	77.38/<0.001
DBP	98.9 (97.9, 99.9)^ab^	95.2 (94.5, 95.9)^c^	88.0 (87.0, 89.0)^abc^	119.26/<0.001

*Normotensives*				
SBP	119.3 (118.5, 120.1)^ab^	116.8 (116.2, 117.5)^c^	117.9 (117.4, 118.4)^abc^	11.35/<0.001
DBP	76.4 (75.8, 77.0)^b^	76.2 (75.8, 76.7)^c^	72.1 (71.7, 72.5)^bc^	128.16/<0.001

SBP: systolic blood pressure, DBP: diastolic blood pressure. ^a^1998–2000 vs. 2007-2008, ^b^1998–2000 vs. 2015, ^c^2007-2008 vs. 2015.

## Data Availability

Data can be made available on reasonable request to the corresponding author.

## Supplementary Materials

Supplementary Materials The supplementary file includes the hypertension treatment algorithm figure.
