# Supplementary figures and images for: Effects of Simplified Antihypertensive Treatment Algorithm on Hypertension Management and Hypertension-Related Death in Resource-Constricted Primary Care Setting between 1997 and 2017
Source: Int J Hypertens. 2021 Jul 13;2021:9920031. doi: 10.1155/2021/9920031 (PMC8294957; doi:10.1155/2021/9920031)

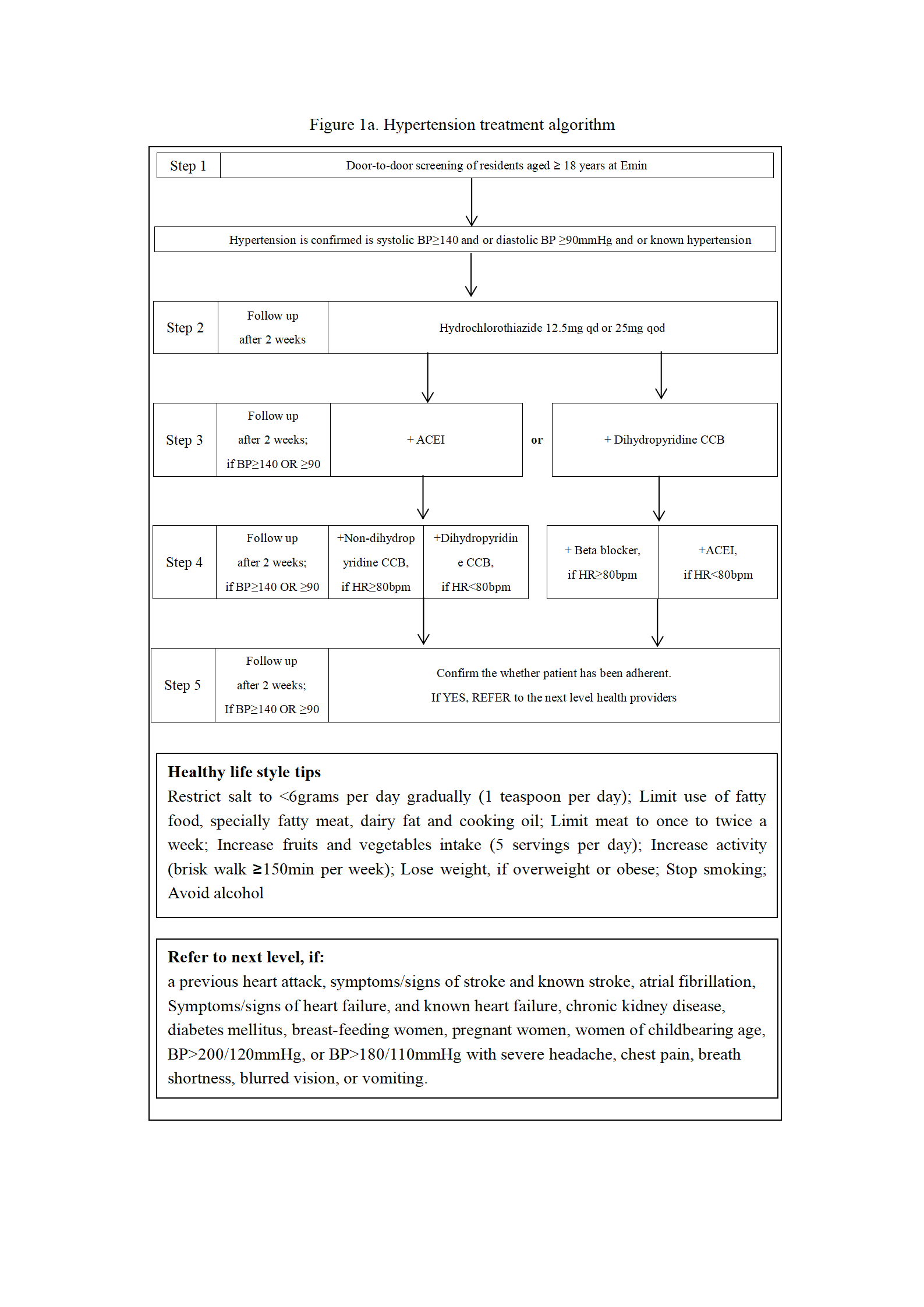

Supplement: Supplementary Materials — The supplementary file includes the hypertension treatment algorithm figure. [file 9920031.f1.doc]
